# Delphi Consensus Recommendations for the Definition of a Severe Bleeding Phenotype and Initiation of Prophylaxis in Patients With Non‐Severe Haemophilia

**DOI:** 10.1111/hae.70259

**Published:** 2026-03-25

**Authors:** Christian Pfrepper, Cihan Ay, Ralf Knöfler, Christoph Königs, Manuela Krause, Wolfgang Miesbach, Johannes Oldenburg, Michael Sigl‐Kraetzig, Rosa Sonja Alesci, Martin Olivieri, for the Standing Committee Hemophilia of the GTH

**Affiliations:** ^1^ Division of Hemostaseology Department of Hematology, Cellular Therapy, Hemostaseology and Infectiology University Hospital Leipzig Leipzig Germany; ^2^ Division of Hematology and Hemostaseology Department of Medicine I Medical University of Vienna Vienna Austria; ^3^ Department of Pediatric Hemostaseology Medical Faculty and University Hospital Carl Gustav Carus of Technische Universität Dresden Dresden Germany; ^4^ Clinical and Molecular Hemostasis Department of Pediatrics and Adolescent Medicine Goethe University University Hospital Frankfurt Frankfurt Germany; ^5^ Department of Hemostaseology Deutsche Klinik für Diagnostik HELIOS Klinik Wiesbaden Germany; ^6^ Medical Clinic II Goethe University Frankfurt Germany; ^7^ Institute of Experimental Hematology and Transfusion Medicine University Hospital Bonn Medical Faculty University of Bonn Bonn Germany; ^8^ Institute For Pediatric Research and Further Education (IPFW) Blaubeuren Germany; ^9^ Pediatric Practice at the South Coagulation Center Blaubeuren Germany; ^10^ MVZ IMD GmbH IMD Blood Coagulation Centre Hochtaunus Bad Homburg Germany; ^11^ Pediatric Thrombosis and Hemostasis Center Pediatric Hemophilia Center Dr. Von Hauner Children´S Hospital LMU Munich Germany

## Abstract

**Introduction:**

Current guidelines recommend prophylaxis for patients with non‐severe haemophilia with severe bleeding phenotype (SBPT) but there is no consensus how to define a SBPT and when to recommend prophylaxis in patients with non‐severe haemophilia.

**Method:**

A Delphi consensus procedure among the members of the Standing Committee Hemophilia of the German, Austrian and Swiss Society of Hemostasis and Thrombosis Research (GTH) was conducted. After defining 41 statements in a steering committee, 26 haemophilia experts participated. Statements were scored on a scale of 1–9, and agreement was defined as a score of ≥ 7. Consensus was defined as ≥ 75%, and strong consensus as ≥ 95% agreement.

**Results:**

After 3 rounds of consent, five major and three minor criteria for SBPT, five recommendations for starting long‐term and six recommendations for starting intermittent prophylaxis were consented. Major criteria included life‐threatening bleeding in critical regions or organs, severe bleeding that occurs spontaneously, repeatedly, or after inadequate trauma, development of haemophilic arthropathy, presence of chronic synovitis, and Hb‐relevant menstrual bleeding. Long‐term prophylaxis should be recommended in patients with a residual factor activity < 3 IU/dL, in patients with a residual activity > 3 IU/dL and a SBPT, following intracranial haemorrhage after assessing the individual risk of recurrence, in cases of comorbidities and medications that cause a permanently increased bleeding tendency, and in the presence of risk factors for severe bleeding or arthropathy.

**Conclusion:**

Consensus was reached on criteria for SBPT and recommendations to initiate prophylaxis in patients with non‐severe haemophilia that can be used in daily practice.

## 1 Introduction

Prophylaxis is the established standard of care for patients with severe haemophilia, as well as for selected patients with moderate haemophilia or other congenital bleeding disorders associated with a severe bleeding phenotype (SBPT) and/or a high risk of spontaneous, life‐threatening bleeding [1]. Increasing evidence indicates that individuals with non‐severe haemophilia may also present with a bleeding phenotype that necessitates prophylactic treatment [2, 3]. However, there is still no internationally accepted definition of an SBPT, and guidance on when prophylaxis should be initiated in this patient population remains limited.

Neither the International Society on Thrombosis and Haemostasis (ISTH) nor the World Federation of Hemophilia (WFH) currently provide a clear and operational definition of SBPT, and existing guidelines do not specify criteria for prophylaxis initiation in patients with non‐severe haemophilia. This lack of consensus hampers clinical decision‐making and contributes to considerable heterogeneity in practice.

To address this unmet need, we conducted a structured Delphi consensus process involving the members of the ‘Standing Committee Hemophilia of the German, Swiss and Austrian Society of Thrombosis and Hemostasis Research (GTH)’. The objective was to achieve consensus‐based recommendations on the definition of a severe bleeding phenotype in patients with non‐severe haemophilia, and clinical criteria for the initiation of prophylaxis in this setting. These recommendations aim to provide a practical framework for clinicians and to serve as a basis for future guideline development for patients with non‐severe haemophilia A (HA) and haemophilia B (HB).

## 2 Methods

In a meeting of the ´Standing Committee Hemophilia of the GTH’ in November 2024, the results of a recently published expert opinion [4] based on a structured literature review were discussed. In order to establish consensus recommendations for patients with non‐severe HA and non‐severe HB, a Delphi consensus procedure was initiated. A group of 10 haemophilia experts from the ´Standing Committee Hemophilia of the GTH’ agreed to participate in the steering committee. The steering committee consisted of haemophilia experts who treat children (*n* = 4), adults (*n* = 5) and both (*n* = 1). Based on the previously published recommendations of a German expert opinion [4], a list of thirteen statements was developed. The statements were divided into 41 questions and sent to all members of the ´Standing Committee Hemophilia of the GTH’ via E‐Mail. All members were allowed to participate in the Delphi process and were asked to express their agreement/disagreement on a scale of 1 (strongly disagree) to 9 (strongly agree). Agreement was defined as a score ≥ 7. Participants were asked to provide explanations in case of disagreement (score ≤ 6) and to give their names for potential follow‐up questions although the provision of names was not mandatory. Consensus was defined as ≥ 75% agreement and strong consensus as ≥ 95% agreement. The responses and comments were evaluated by the steering committee after each round. A total of 26 haemophilia experts from the ´Standing Committee Hemophilia of the GTH’ participated in the first, 23 in the second, and 22 in the third round.

After the first round, the first four major criteria were consented as suggested. In an open question, participants of the Delphi process were asked for additional suggestions for Major criteria. The fifth major criterion was consented in the second round.

## 3 Consensus Statements

### 3.1 Definition of a Severe Bleeding Phenotype

#### 3.1.1 Definition of Major and Minor Criteria

1. A severe bleeding phenotype is defined by major and minor criteria.
  Consensus: 96.2%.
2. The presence of one major criterion defines a severe bleeding phenotype.
  Consensus: 84.6%.
3. The presence of ≥ 2 minor criteria define a severe bleeding phenotype even without a major criterion.
  Consensus: 84.6%.

#### 3.1.2 Major Criteria

1. Major criterion 1 is:
  Life‐threatening bleeding in critical regions or organs that occurred spontaneously or after minor trauma, such as
  - Intracranial, intraocular, retroperitoneal, intraarticular, pericardial or
  - Muscle bleeding with compartment syndrome or
  - Bleeding leading to a loss in haemoglobin ≥ 2.0 g/dL (1.24 mmol/L) or red blood cell transfusion
  Consensus: 92.3%.
  - When applying Major criterion 1, temporary causes of bleeding that can be treated should be taken into account. Consensus: 92.3%.
  - After appropriate and sustained treatment of a temporary cause of bleeding according to Major criterion 1, the bleeding is no longer defining for a severe bleeding phenotype. Consensus: 88.6%.
2. Major criterion 2 is:
  Severe bleeding that occurs spontaneously, repeatedly, or after inadequate trauma, such as muscle bleeding or bleeding with consequences for function and structure.
  Consensus: 96.2%.
3. Major criterion 3 is:
  Development of haemophilic arthropathy.
  Consensus: 96.2%.
4. Major criterion 4 is:
  Presence of chronic synovitis.
  Consensus: 88.6%.
5. Major criterion 5 is:
  Hb‐relevant menstrual bleeding despite exhausting conservative measures such as tranexamic acid, DDAVP, and/or hormone therapy.
  Consensus: 91.3%.

#### 3.1.3 Minor Criteria

1. Minor criterium 1 is:
  Repeated spontaneous extensive hematomas requiring substitution with factor concentrates. Cconsensus 100%
2. Minor criterium 2 is:
  - A positive family history for a severe bleeding phenotype.
    Consensus 84.6%
  - A positive family history for life‐threatening bleeding.
    Consensus 80.8%
  - One criterion is sufficient for diagnosing minor criterion 2.
    Consensus 78.3%
3. Minor criterion 3 is:
  Repeated bleeding following inadequate trauma that does not impair function and structure but may indicate a potential structure‐impairing bleeding.
  Consensus 80.8%.

#### 3.1.4 Statements Regarding the Severe Bleeding Phenotype

1. The complete remission of synovitis after a single episode of bleeding under rehabilitation and prophylaxis does not necessarily indicate a SBPT and justify long‐term prophylaxis. Consensus: 76.9%
2. A spontaneous bleeding tendency is a sign of a SBPT; therefore, prophylaxis should be evaluated. Consensus 92.3%
3. When evaluating a spontaneous bleeding tendency, lifestyle, age, physical activity, and occupation should be taken into consideration. Consensus 96.2%
4. A positive family history may indicate a similar bleeding phenotype in family members, although a different phenotype is possible. Consensus 88.5%.

#### 3.1.5 Comments

##### 3.1.5.1 Major Criteria

Participants discussed regarding major criterion 1 that bleeding resulting in a Hb loss > 2 g/dL or requiring red blood cell transfusion may not apply to children below the weight of 30 kg and that it should be applied with caution in patients with trauma, GI‐bleeding and in patients undergoing surgery.

Some participants noted that recurrent minor or even subclinical bleeding may be causative for haemophilic arthropathy and chronic synovitis (Major criteria 3 and 4) and that other causes than bleeding for chronic synovitis like rheumatoid arthritis should be excluded.

##### 3.1.5.2 Minor Criteria

In the first round, minor criterion 1 defined as ‘repeated spontaneous hematoma’ failed to reach consensus. In the second round three other options were given but no statement reached consensus. After discussing the comments of the participants in the steering committee, only one statement was given in third round and reached consensus from all participants.

A family history of a haemophilic arthropathy failed to reach consensus in the first round (consensus 73.1%) and was excluded.

##### 3.1.5.3 Additional Statements

Some participants have noted that a family history of a severe bleeding phenotype or life‐threatening bleeding is not always indicative of a patient's phenotype, as accompanying circumstances such as occupation, physical activity, and haemophilia therapy may have contributed to the phenotype in the relative.

### 3.2 Recommendations for Starting Prophylaxis

#### 3.2.1 Long‐Term Prophylaxis

1. Long‐term prophylaxis should be recommended at a residual factor activity < 3 IU/dL. Consensus 84.5%
2. Long‐term prophylaxis should be recommended at residual factor activity > 3 IU/dL and the presence of a SBPT. Consensus 88.5%
3. Following intracranial haemorrhage, the initiation of long‐term prophylaxis is justified After assessing the individual risk of recurrence. Consensus 100%
4. Long‐term prophylaxis should be recommended in cases of comorbidities and medications that cause a permanently increased bleeding tendency. Consensus 92.3%
5. Long‐term prophylaxis should be recommended in the presence of risk factors for severe bleeding or arthropathy. Consensus 84.6%
6. The preferences of parents and patients should be taken into account when initiating long‐term prophylaxis. Consensus 100%

#### 3.2.2 Intermittent Prophylaxis

1. Intermittent prophylaxis should be recommended regardless of residual factor activity in the presence of comorbidities and medications that cause a temporarily increased bleeding tendency. Consensus 88.5%
2. Intermittent prophylaxis can be recommended regardless of residual activity in connection with physical activity with an increased risk of bleeding. Consensus 78.3%
3. Intermittent prophylaxis should be initiated regardless of residual activity in the context of surgical procedures with an increased perioperative bleeding risk. Consensus 96.2%
4. Intermittent prophylaxis can be recommended regardless of residual activity During rehabilitation measures. Consensus 84.6%
5. Intermittent prophylaxis can be recommended regardless of residual activity in women with haemophilia who suffer from menorrhagia. Consensus 84.6%
6. Intermittent prophylaxis can be recommended regardless of residual activity in treatment‐refractory recurrent mucosal bleeding. Consensus 91.3%

#### 3.2.3 Statements for Starting Prophylaxis

1. Comorbidities associated with a permanently increased bleeding risk include liver cirrhosis, epilepsy, thrombocytopenia, intracranial aneurysms, angiodysplasia, cancer, chronic inflammatory bowel diseases, or patients with an additional congenital or acquired bleeding tendency. Consensus 84.0%
2. There are fluid transitions Between intermittent and permanently elevated bleeding risk. Consensus 92.0%
3. The indication for prophylaxis should be reevaluated regularly, taking into account factors such as growth in children, and changes in lifestyle and occupation. Consensus 96.0%
4. The initiation of intermittent prophylaxis is based on an individual decision by the practitioner and the patient. Consensus 95.7%

#### 3.2.4 Comments

Long‐term prophylaxis has been defined as the initiation of prophylaxis that is to be continued indefinitely, in contrast to intermittent prophylaxis, which is initiated only for a specific period of time. In the first round, statements regarding the initiation of prophylaxis were phrased as ‘should or can be initiated’. Some participants noted that these statements should be rephrased to ‘should or can be recommended’. Re‐phrasing these statements reached 82.6% consensus in the second round.

##### 3.2.4.1 Long‐Term Prophylaxis

Although the residual activity for initiating long‐term prophylaxis was consented to be < 3 IU/dL, some participants have noted that the threshold should be < 5 IU/dL as most patients with severe haemophilia will have at least a trough level of 5 IU/dL or more. On the other hand, some participants argued that patients with 2.8 IU/d without bleeding may not necessarily need long‐term prophylaxis. In addition, residual factor activity and bleeding tendency should be taken into account, when comorbidities and medications indicate the need for long‐term prophylaxis. Apart from that, the lower residual factor activity should be considered in case of discrepancies between values measured by one stage and chromogenic assay. Finally, the statement that the preferences of parents and patients should be taken into account when initiating long‐term prophylaxis reached 100% consensus. When implementing this in clinical practice, consensus should be reached by shared decision making.

##### 3.2.4.2 Intermittent Prophylaxis

Comparable to the initiation of long‐term prophylaxis, residual factor activity and individual bleeding risk should be evaluated before recommending intermittent prophylaxis. This applies especially to anticoagulants, type of physical activity, surgical procedure or rehabilitation measures.

## 4 Discussion

This Delphi consensus represents the first structured effort to define a SBPT and to provide harmonised, evidence‐informed recommendations for initiating prophylaxis in patients with non‐severe HA and HB. Building on the expert proposals published by Pfrepper et al. [4], the present consensus translates these concepts into an operational, consensus‐based framework validated through a structured, multi‐round Delphi process among German, Swiss, and Austrian haemophilia experts.

### 4.1 Definition of a Severe Bleeding Phenotype

The consensus confirmed that an SBPT can be defined by the presence of at least one major or two minor criteria. Compared with the earlier expert opinion [4], these criteria were refined and validated in a systematic manner. Importantly, Hb‐relevant menstrual bleeding despite optimal conservative therapy (e.g., tranexamic acid, DDAVP, hormonal treatment) was added as a new major criterion, acknowledging that symptomatic women and women with haemophilia may also present with clinically significant bleeding requiring prophylaxis [5, 6]. The consensus further emphasised that temporary and treatable causes of bleeding—such as trauma, gastrointestinal lesions, or perioperative haemorrhage—should not automatically qualify as defining events for an SBPT. Similarly, chronic synovitis following a single episode that resolves completely under appropriate management should not be regarded as indicative of a persistent severe phenotype. These distinctions highlight the importance of individualised clinical judgement and reflect the growing recognition that the bleeding phenotype is influenced by comorbidities, lifestyle, and activity rather than factor levels alone [3, 7]. To emphasise the importance of clinical judgement and to make our statements applicable to a broader spectrum of physicians, we did not recommend specific definitions for haemophilic arthropathy or chronic synovitis based on the use of MRI or ultrasound.

### 4.2 Comparison With Previous Expert Proposals

The present recommendations are consistent with those of Pfrepper et al. [4], who proposed clinically relevant major and minor criteria without introducing a formal scoring system. However, the Delphi consensus strengthens this framework by achieving quantitative expert agreement (≥ 75% for consensus, ≥ 95% for strong consensus) and by refining several details. For example, family history of haemophilic arthropathy was excluded due to insufficient agreement, whereas family history of an SBPT or life‐threatening bleeding was maintained as a minor criterion. This modification recognises that genetic, environmental, and therapeutic factors may all modulate the bleeding phenotype. [8, 9] Moreover, the consensus introduces menstrual and mucosal bleeding aspects that were not explicitly addressed in the 2024 expert opinion. This extension broadens the applicability of the SBPT concept across the full spectrum of haemophilia presentations, including women with factor deficiency or carrier status [1, 6].

### 4.3 Recommendations for Prophylaxis Initiation

Consistent with prior expert recommendations [4, 10], the panel agreed that long‐term prophylaxis should generally be initiated at a residual factor activity < 3 IU/dL, irrespective of the bleeding phenotype. For patients with activity levels above this threshold, prophylaxis is recommended in the presence of an SBPT or persistent risk factors for bleeding that are independent of factor activity alone. These include haemophilic arthropathy, chronic synovitis and target joint bleeding, as well as comorbidities and medications that persistently increase bleeding tendency (such as liver disease, thrombocytopenia or antithrombotic therapy). This is consistent with recommendations from the ISTH [11] and an Italian expert panel [12]. This approach is supported by data from the MoHem [13] and THUNDER [14] studies, which showed that individuals with moderate haemophilia and baseline factor levels ≤ 3 IU/dL exhibit joint outcomes and bleeding rates comparable to those of patients with severe disease. In addition, the PROPEL study has shown that, increasing FVIII trough levels in patients with severe HA from 1–3 IU/dL to 8–12 IU/dL resulted in a lower ABR and a higher proportion of patients with zero bleeds particularly in those with a short half‐life [15, 16].

The recommendations also clarify the indications for intermittent prophylaxis, which can be applied regardless of residual activity in situations with a temporary bleeding risk—such as intensive physical activity, surgical procedures, rehabilitation, or refractory mucosal bleeding. Including treatment‐refractory mucosal bleeding and menorrhagia as specific indications represents an important evolution of the prophylactic concept, addressing common but previously under‐recognised clinical scenarios [6, 17, 18].

### 4.4 Clinical Implications and Limitations

By providing consensus‐based, operational criteria, this report helps to bridge the gap between laboratory classification and clinical management of non‐severe haemophilia. The recommendations support individualised assessment that integrates factor levels, bleeding history, comorbidities, and lifestyle. They further contribute to standardizing decisions across centres and to reducing variability in prophylaxis initiation observed in recent registries [19, 20]. However, the findings should be interpreted considering certain limitations. While the consensus thresholds (≥ 75% agreement) provide methodological robustness, the statements remain based on expert interpretation rather than prospective validation. In addition, the consensus reflects expert opinion from a defined regional cohort and may not fully represent international practice diversity. Particularly in countries with limited access to factor supply our recommendations may not be implementable. In addition, this Delphi consensus was not intended to be an evidence‐based guideline. Our statements are based on clinical experience from an expert board of a national medical society and did not follow a pre‐defined criteria like the GRADE methodology [21]. Therefore, we cannot exclude that some relevant aspects may not been have addressed and that potential conflicts of interests of some participants may have influenced the results. Future international Delphi rounds and longitudinal studies should evaluate the predictive value of the proposed SBPT criteria and their impact on patient‐reported outcomes and joint health. As a consequence, evidence‐based guidelines from international societies like the ISTH and WFH may be established.

## 5 Conclusion

This Delphi consensus establishes a clinically applicable framework for defining a severe bleeding phenotype and for guiding the initiation of prophylaxis in non‐severe haemophilia. By achieving a high level of expert agreement, the consensus complements and extends previous expert opinion [4, 11, 12] and provides a basis for future international guideline development. Its implementation may contribute to a more individualised and standardised management of patients with non‐severe haemophilia for improving outcomes and quality of life.

## Funding

The authors have nothing to report.

## Ethics Statement

Not Applicable

## Conflicts of Interest

The authors declare no conflicts of interest.

## Data Availability

The data that support the findings of this study are available from the corresponding author upon reasonable request
